# Feasibility and effectiveness of afatinib for poor performance status patients with *EGFR*-mutation-positive non-small-cell lung cancer: a retrospective cohort study

**DOI:** 10.1186/s12885-021-08587-w

**Published:** 2021-07-27

**Authors:** Chiao-En Wu, Ching-Fu Chang, Chen-Yang Huang, Cheng-Ta Yang, Chih-Hsi Scott Kuo, Ping-Chih Hsu, John Wen-Cheng Chang

**Affiliations:** 1 Department of Internal Medicine, Division of Hematology-Oncology, Chang Gung Memorial Hospital at Linkou, Chang Gung University College of Medicine, No. 5, Fu-Hsing Street, Kwei-Shan District, Taoyuan City, 333 Taiwan; 2 Department of Thoracic Medicine, Division of Thoracic Oncology, Chang Gung Memorial Hospital at Linkou, Chang Gung University College of Medicine, No. 5, Fu-Hsing Street, Kwei-Shan District, Taoyuan City, 333 Taiwan

**Keywords:** Lung cancer, Afatinib, EGFR, TKI, Performance status

## Abstract

**Background:**

Afatinib is one of the standard treatments for patients with epidermal growth factor receptor (*EGFR*)-mutated non-small-cell lung cancer (NSCLC). However, data on the use of afatinib in patients with poor performance status (PS ≥ 2) are limited. This study aimed to retrospectively review the clinical outcomes and safety of afatinib treatment in *EGFR*-mutation-positive (EGFRm+) NSCLC patients with PS ≥ 2.

**Methods:**

The data for 62 patients who were treated at Linkou Chang Gung Memorial Hospital from January 2010 to August 2019 were retrospectively reviewed. Patients’ clinicopathological features were obtained, and univariate and multivariate analyses were performed to identify possible prognostic factors. Data on adverse events were collected to evaluate general tolerance for afatinib therapy.

**Results:**

Until February 2020, the objective response rate, disease control rate, median progression-free survival (PFS), and overall survival (OS) were 58.1% (36/62), 69.4% (43/62), 8.8 months, and 12.9 months, respectively. The absence of liver metastasis (PFS: *p* = 0.044; OS: *p* = 0.061) and good disease control (*p* < 0.001 for PFS and OS) were independent favorable prognostic factors for PFS and OS. Bone metastasis (*p* = 0.036) and dose modification (reduction/interruption, *p* = 0.021) were predictors of disease control.

**Conclusion:**

Afatinib demonstrated acceptable efficacy and safety in the current cohort. This study provided evidence to support the use of afatinib as a first-line treatment in EGFRm+ NSCLC patients with poor PS.

## Background

Activating mutations in the epidermal growth factor receptor (*EGFR*) gene in non-small-cell lung cancer (NSCLC) lead to an increase in growth factor signaling activity and susceptibility to tyrosine kinase inhibitors (TKIs) [1, 2]. First-generation EGFR-TKIs, such as gefitinib and erlotinib, have become the first-line treatment for patients with *EGFR*-mutation-positive (EGFRm+) NSCLC since 2010 [3–6]. Later, afatinib, a second-generation EGFR TKI, which serves as a pan-human EGFR(HER) family inhibitor that irreversibly binds to EGFR, was approved for the treatment of EGFRm+ NSCLC, based on the results of pivotal randomized clinical studies (LUX-Lung 3, LUX-Lung 6, and LUX-Lung 7) [7–9]. Although osimertinib, a third-generation EGFR TKI, recently demonstrated superior survival outcomes compared with first-generation EGFR-TKIs (either gefitinib or erlotinib) as first-line treatment [10, 11], afatinib remains widely used in daily practice due to its cost-effectiveness.

Although patients are typically treated based on the outcomes of clinical trials, clinical trials typically apply restrictive inclusion and exclusion criteria, which cannot be completely applied to real-world practice; therefore, real-world experiences could provide additional information regarding the effectiveness of afatinib treatment in patients with EGFRm+ NSCLC [12–15], particularly in those patients with the types of clinicopathological features that were excluded from previous clinical studies, such as uncommon *EGFR* mutations, brain metastases, advanced age, or poor performance status (PS) [16]. PS is an important prognostic and predictive factor in most cancer treatments. Previous clinical trials for afatinib only enrolled patients with good Eastern Cooperative Oncology Group (ECOG) PS scores of 0 or 1; therefore, the feasibility of afatinib in patients with poor PS remains unknown, although we treat these patients based on the outcomes of these clinical trials. In real-world cohorts, patients with PS ≥ 2 account for 10–20% of all cases, and the number of patients with this score is limited [12, 13, 17–19]. Therefore, this study aimed to investigate the feasibility and efficacy of afatinib in patients with EGFRm+ NSCLC and poor PS (PS ≥ 2).

## Methods

### Data collection

Data for all study patients were obtained from the Chang Gung Research Database [20], which is an integrated and comprehensive database consisting of multi-institutional standardized electronic medical records from all Chang Gung Memorial Hospitals (CGMHs) in Taiwan, including information from the cancer registry. Data for patients were obtained from the cancer registry for Linkou CGMH from January 2010 to August 2019.

### Eligibility and exclusion criteria

Patients who were diagnosed with advance (Stage IIIB and Stage IV, based on the American Joint Committee on Cancer staging system 7th edition) lung cancer [based on the International Disease Classification, 10th revision, Clinical Modification (ICD-10-CM) codes of C3400–C3492], with PS ≥ 2, *EGFR* mutation, and who were treated with EGFR-TKIs as first-line treatment, without prior systemic treatment, were enrolled in the study. The *EGFR* mutation status of the tumors was retrospectively reviewed. Patients with single-nucleotide polymorphisms without activating mutation (*n* = 3) and those with a de novo T790M mutation (*n* = 7) were excluded. Finally, a total of 246 patients treated with various EGFR-TKIs as first-line treatment including 62 patients treated with afatinib were included in this study. This study was approved by the Institutional Review Board of CGMH (201901395B0). Patient consent to participate was not required due to the retrospective nature of this study.

### Patients’ characteristics and treatment course

The data of 62 patients who received afatinib as first-line treatment at Linkou CGMH were retrospectively reviewed. The clinicopathological features, including age, sex, body weight, height, smoking history, PS, tumor involvement, *EGFR* mutation (19del, L858R, or uncommon mutation), starting dose of afatinib, dose modification (reduction/interruption) of afatinib, tumor response, adverse events (AEs), and subsequent treatment were obtained. The last follow-up time point in the study was February 2020.

### Treatment and response evaluation

The patients were treated with afatinib at a starting dose of either 30 or 40 mg, administered once daily until disease progression or intolerable toxicity. The dose and schedule of afatinib were adjusted by individual physicians based on the patients’ clinical condition and AEs due to treatment. Tumor response was evaluated by chest radiography, computed tomography, or positron emission tomography. The Response Evaluation Criteria in Solid Tumors 1.1 criteria were used to evaluate the best tumor response. The best clinical tumor response was recorded as complete response (CR), partial response (PR), stable disease (SD), or progressive disease (PD). Any tumor response that was not assessed before death or discontinuation due to intolerance was recorded as “not assessed” (NA). Progression-free survival (PFS) was defined as the duration from the first day of afatinib treatment until the first radiological evidence of disease progression, the last dose of afatinib, death, or the latest follow-up time point. Those patients who did not experience progression nor death were censored during PFS analysis. Overall survival (OS) was defined as the duration from the first day of afatinib treatment until the date of death or last follow-up. The data for patients who did not experience death were censored when survival curves were analyzed. The objective response rate (ORR), expressed in percentage, was taken as the sum of CR and PR; the disease control rate (DCR), expressed in percentage, was taken as the sum of CR, PR, and SD.

### Adverse events

Data about AEs were collected from electronic medical records and graded according to the National Cancer Institute Common Terminology Criteria for Adverse Events, version 4.0. All grades of AEs and severe AEs (Grades 3/4) were collected. Dose reductions, interruptions, or withdrawals due to the occurrence of AEs were recorded.

### Statistical analysis

The PFS and OS were estimated using the Kaplan-Meier method and their prognostic factors were compared using the log-rank test. Univariate analysis was performed to evaluate possible prognostic factors including age, sex, staging, *EGFR* mutation status, PS, smoking history, body mass index (BMI), body surface area (BSA), tumor involvements, and clinical tumor response. Multivariate analysis was performed to evaluate independent prognostic factors. The results are presented as the hazard ratio (HR) and 95% confidence interval (CI) from Cox regression analyses. IBM SPSS Statistics for Windows (Version 22.0, Armonk, NY, USA) was used to perform all statistical analyses, and *p* < 0.05 was considered significant. We used the R package “survival” and “survminer” to plot survival curves and generate Cox proportional hazard models.

## Results

### Patient characteristics

In this study, a total of 62 EGFRm+ NSCLC patients with ECOG PS ≥ 2 who were treated with first-line afatinib as a systemic treatment were examined. The patients’ ages ranged from 36.6 to 89.0 years, with a median age of 66.7 years, 22 (35.5%) were men, and 40 (64.5%) were women, and all patients were Asians. All patients had Stage IV disease, except one who had Stage IIIb disease, according to the American Joint Committee on Cancer staging system 7th edition. Fifty-two patients (85.2%) had no smoking history. The tumor histology for all patients was adenocarcinoma. Thirty-eight (61.3%) patients had a PS of 2, whereas 24 (38.7%) patients had a PS > 2. The *EGFR* mutation identified most frequently were L858R (*n* = 30, 48.4%) and 19del (*n* = 25, 40.3%), and 7 (11.3%) patients had uncommon *EGFR* mutations. In terms of tumor involvement, bone was the most common metastatic site (51.6%), followed by lung (43.5%) and brain (43.5%). The starting dose for 39 (62.9%) patients was 40 mg afatinib daily, whereas the starting dose for 23 (37.1%) patients was 30 mg afatinib daily (Table 1).

**Table 1:** Patient’s characteristics and associations with clinical response

Characteristics	Total (*N* = 62)	Response		*p*-value
		CR/PR/SD (*N* = 47)	PD/NA (*N* = 15)	
Age, median (IQR)	66.7 (18.1)	65.1 (19.5)	71.2 (16.1)	0.42
≤ 65	27 (43.5%)	23 (85.2%)	4 (14.8%)	0.13
> 65	35 (56.5%)	24 (68.6%)	11 (31.4%)	
Sex				
Male	22 (35.5%)	16 (72.7%)	6 (27.3%)	0.675
Female	40 (64.5%)	31 (77.5%)	9 (22.5%)	
Stage				
Stage 3B	1 (1.6%)	1 (100.0%)	0 (0.0%)	0.569
Stage 4	61 (98.4%)	46 (75.4%)	15 (24.6%)	
Smoking status^a^				
Smoker	9 (14.8%)	8 (88.9%)	1 (11.1%)	0.36
Never smoker	52 (85.2%)	39 (75.0%)	13 (25.0%)	
Histology				
Adenocarcinoma	62 (100.0%)	47 (75.8%)	15 (24.2%)	–
Performance status, PS				
PS 2	38 (61.3%)	28 (73.7%)	10 (26.3%)	0.623
PS 3/4	24 (38.7%)	19 (79.2%)	5 (20.8%)	
Mutation				
L858R	30 (48.4%)	24 (80.0%)	6 (20.0%)	0.097
19del	25 (40.3%)	20 (80.0%)	5 (20.0%)	
Uncommon	7 (11.3%)	3 (42.9%)	4 (57.1%)	
Starting dose				
40 mg	39 (62.9%)	31 (79.5%)	8 (20.5%)	0.378
30 mg	23 (37.1%)	16 (69.6%)	7 (30.4%)	
Metastatic sites				
Lung				
Yes	27 (43.5%)	19 (70.4%)	8 (29.6%)	0.38
No	35 (56.5%)	28 (80.0%)	7 (20.0%)	
Liver				
Yes	14 (22.6%)	10 (71.4%)	4 (28.6%)	0.664
No	48 (77.4%)	37 (77.1%)	11 (22.9%)	
Brain				
Yes	27 (43.5%)	19 (70.4%)	8 (29.6%)	0.38
No	35 (56.5%)	28 (80.0%)	7 (20.0%)	
Bone				
Yes	32 (51.6%)	28 (87.5%)	4 (12.5%)	0.026
No	30 (48.4%)	19 (63.3%)	11 (36.7%)	
Pleura				
Yes	21 (33.9%)	16 (76.2%)	5 (23.8%)	0.96
No	41 (66.1%)	31 (75.6%)	10 (24.4%)	
Adrenal gland				
Yes	4 (6.5%)	3 (75.0%)	1 (25.0%)	0.969
No	58 (93.5%)	44 (75.9%)	14 (24.1%)	
Distant lymphadenopathy				
Yes	6 (9.7%)	4 (66.7%)	2 (33.3%)	0.582
No	56 (90.3%)	43 (76.8%)	13 (23.2%)	
Dose reduction/interruption				
Yes	23 (37.1%)	21 (91.3%)	2 (8.7%)	0.029
No	39 (62.9%)	26 (66.7%)	13 (33.3%)	
Discontinuation, AE-related				
Yes	8 (12.9%)	5 (62.5%)	3 (37.5%)	0.346
No	54 (87.1%)	42 (77.8%)	12 (22.2%)	
BMI, median (IQR)	23.11 (4.50)	22.68 (3.75)	25.98 (8.46)	0.059
BSA, median (IQR)	1.57 (0.17)	1.57 (0.17)	1.60 (0.20)	0.48

*IQR* interquartile range, *CR* complete response, *PR* partial response, *SD* stable disease, *PD* progressive disease, *NA* not assessed, *BMI* body mass index, *BSA* body surface area, *AE* adverse events

^a^There is one missing data point on smoking status

**Fig. 1:**
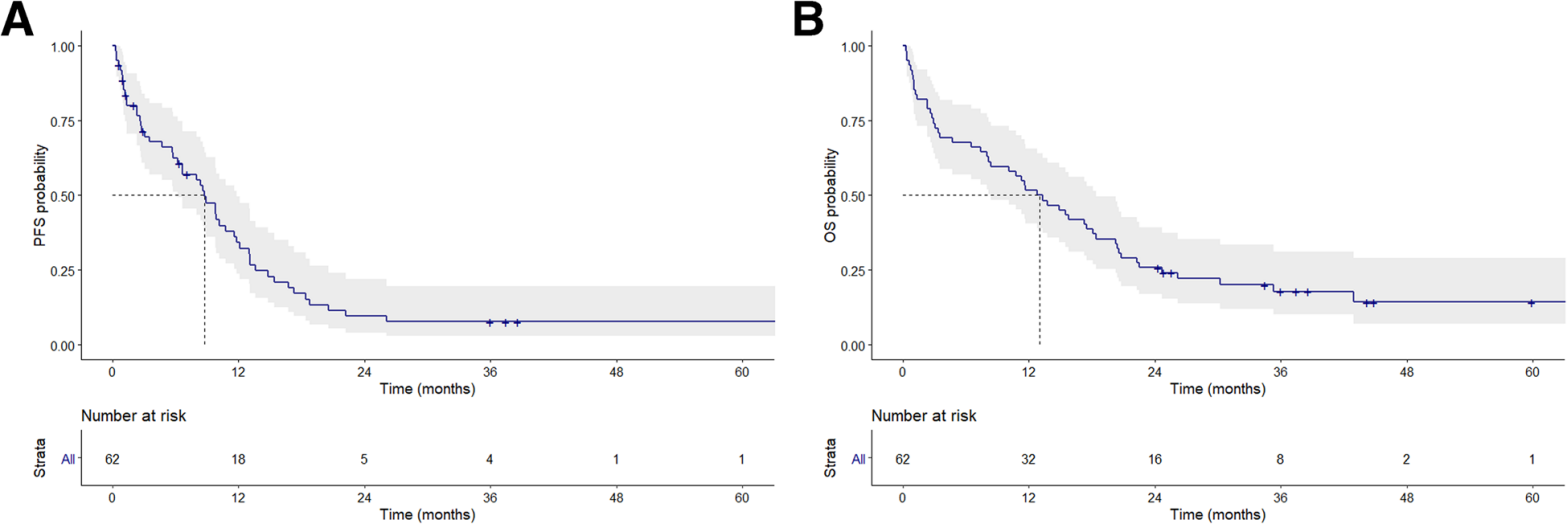
Kaplan-Meier survival curves for PFS (**A**) and OS (**B**) among patients treated with afatinib. PFS, progression-free survival; OS, overall survival

By the end of February 2020, the follow-up time ranged from 0.3 to 64.5 months, with a median follow-up time of 13.1 months. The median PFS (mPFS) and median OS (mOS) were 8.8 months (95% CI: 6.78–10.77 months) and 12.9 months (95% CI: 8.35–17.35 months), respectively (Fig. 1). The ORR was 58.1% (*n* = 36) and the DCR was 69.4% (*n* = 43).

### Prognostic factors for PFS

**Fig. 2:**
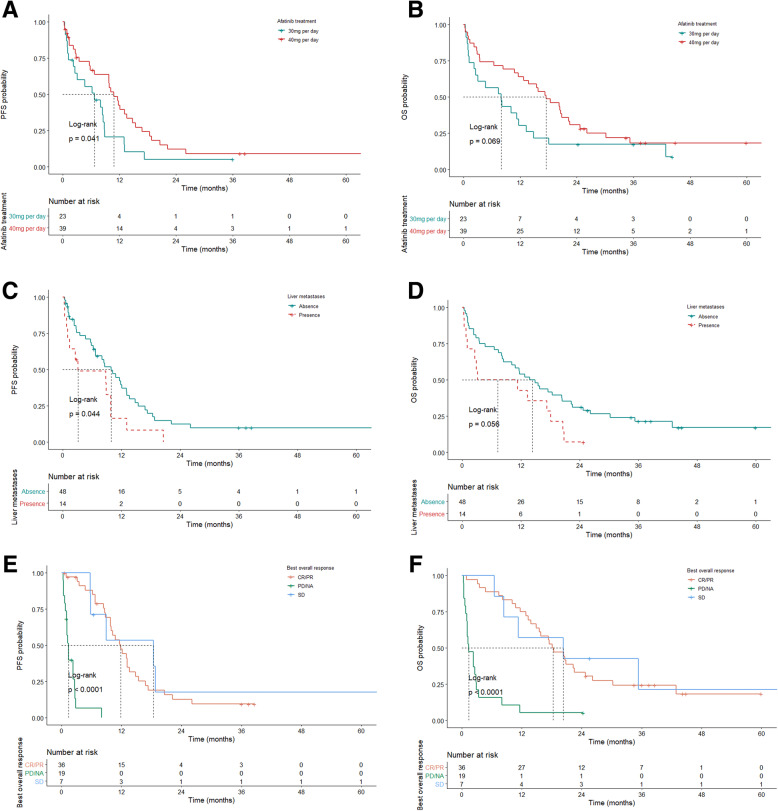
Kaplan-Meier survival curves for PFS (**A, C**, and **E**) and OS (**B, D**, and **F**) among patients stratified according to prognostic factors: starting dose of afatinib (**A** and **B**), liver metastases (**C** and **D**), and clinical tumor responses (**E** and **F**). PFS, progression-free survival; OS, overall survival; CR, complete response; PR, partial response; SD, stable disease, PD progressive disease; NA, not assessed

**Fig. 3:**
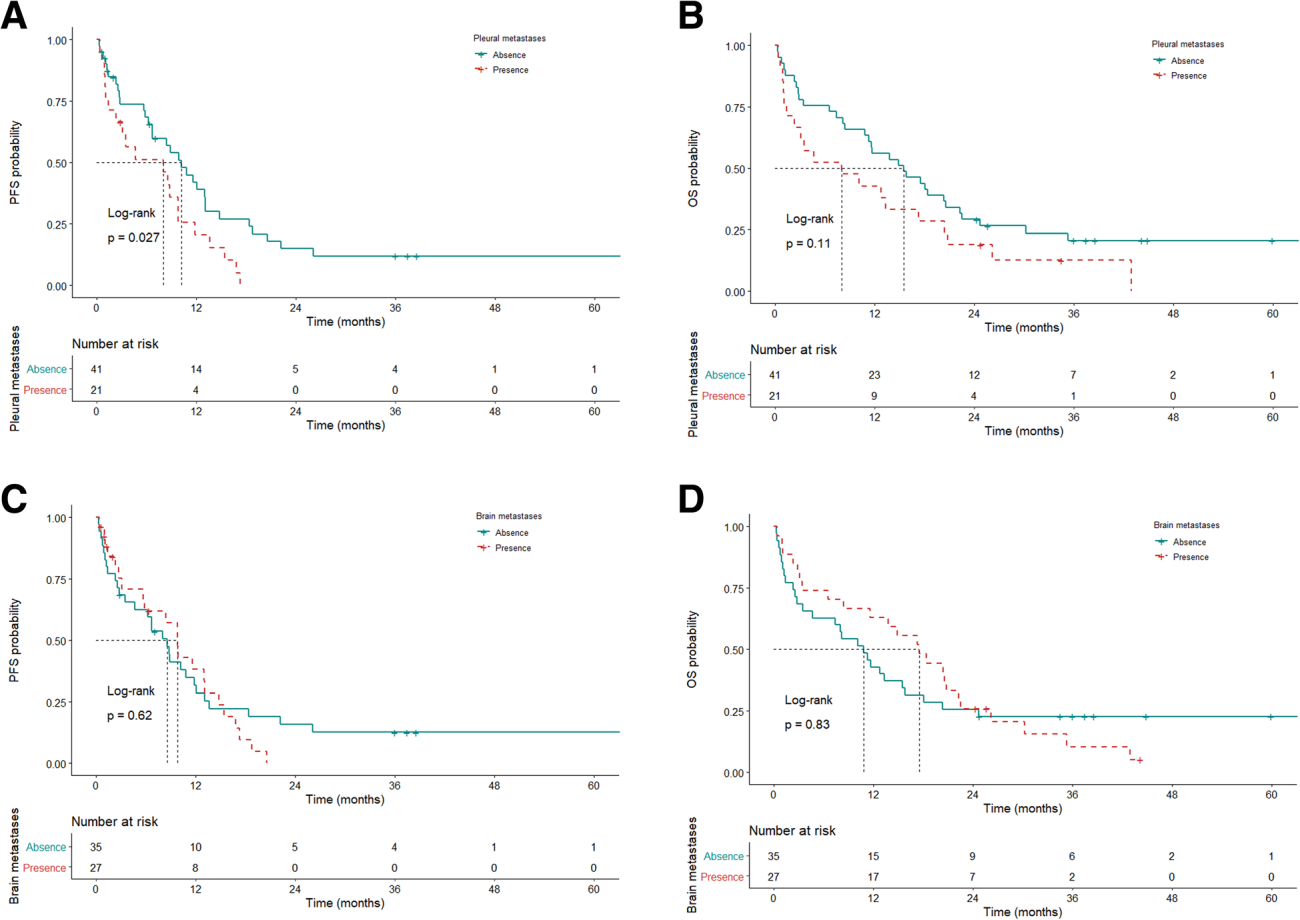
Kaplan-Meier survival curves for PFS (**A** and **C**) and OS (**B** and **D**) among patients stratified according to pleural metastasis (**A** and **B**) and brain metastasis (**C** and **D**). PFS, progression-free survival; OS, overall survival

A univariate analysis was performed to identify possible prognostic factors for PFS in patients treated with afatinib. Patients who received a starting dose of 40 mg (vs. 30 mg, mPFS: 10.8 vs. 6.7 months, HR: 0.55, 95% CI: 0.31–0.98, *p* = 0.043) had favorable PFS (Fig. 2A). Patients who experienced liver metastases (vs. no liver metastases, mPFS: 3.1 vs. 9.9 months, HR: 1.94, 95% CI: 1.02–3.69, *p* = 0.044; Fig. 2C) and pleural metastases (vs. no pleural metastases, mPFS: 8.1 vs. 10.2 months, HR: 1.92, 95% CI: 1.07–3.45, *p* = 0.03; Fig. 3A) had unfavorable PFS. Patients who achieved CR/PR (vs. PD/NA, mPFS: 11.8 vs. 1.4 months, HR: 0.05, 95% CI: 0.02–0.13, *p* < 0.001) or SD (vs. PD/NA, mPFS: 18.4 vs. 1.4 months, HR: 0.04, 95% CI: 0.01–0.14, *p* < 0.001) showed better PFS than those with a tumor response of PD/NA (Fig. 2E and Table 2).

**Table 2:** Univariate and multivariate analysis of prognostic factors in patients (PFS)

Parameters	Univariate					Multivariate		
	Median (months)	95% CI	HR	95% CI	*p-*value	HR	95% CI	*p-*value
Age								
≤ 65	9.9	7.8–12.0	0.95	0.54–1.66	0.862			
> 65	8.1	1.2–15.0						
Sex								
Male	11.6	7.2–16.1	0.66	0.37–1.16	0.15			
Female	6.7	3.2–10.2						
Smoking status^a^								
Smoker	14.8	10.1–19.5	0.54	0.25–1.16	0.113			
Never smoker	8.1	5.5–10.6						
Performance status, PS								
PS 2	9.9	8.4–11.4	0.94	0.52–1.69	0.826			
PS 3/4	6.2	5.0–7.5						
Mutation					0.446			
L858R	8.4	5.2–11.5	0.77	0.33–1.80	0.548			
19del	10.2	7.3–13.1	0.59	0.25–1.41	0.235			
Uncommon	3.5	0.5–6.6						
Starting dose								
40 mg	10.8	8.6–13.1	0.55	0.31–0.98	0.043	0.92	0.49–1.75	0.806
30 mg	6.7	1.9–11.5						
Metastatic sites								
Lung								
Yes	8.1	5.5–10.6	1.41	0.81–2.46	0.222			
No	9.9	8.5–11.3						
Liver								
Yes	3.1	0.0–13.3	1.94	1.02–3.69	0.044	2.17	1.11–4.26	0.023
No	9.9	6.5–13.3						
Brain								
Yes	9.8	7.6–12.0	1.16	0.66–2.04	0.615			
No	8.6	5.8–11.5						
Bone								
Yes	9.8	8.1–11.5	0.65	0.38–1.14	0.133			
No	5.9	1.4–10.2						
Pleura								
Yes	8.1	0.8–15.3	1.92	1.07–3.45	0.03	1.58	0.84–2.98	0.157
No	10.2	6.8–13.6						
Adrenal gland								
Yes	8.6	0.0–21.1	1.13	0.35–3.64	0.842			
No	8.9	5.4–12.3						
Distant lymphadenopathy								
Yes	6.7	0.0–15.9	1.91	0.74–4.94	0.183			
No	8.9	7.0–10.8						
Dose reduction/interruption								
Yes	11.6	9.0–14.3	0.65	0.36–1.16	0.143			
No	6.2	0. 0–12.7						
Discontinuation, AE-related								
Yes	6.7	0.6–12.8	0.64	0.20–2.07	0.457			
No	8.9	6.9–10.8						
Clinical tumor response					< 0.001			< 0.001
CR/PR	11.8	9.2–14.4	0.05	0.02–0.13	< 0.001	0.05	0.02–0.13	< 0.001
SD	18.4	4.7–32.1	0.04	0.01–0.14	< 0.001	0.05	0.01–0.19	< 0.001
PD/NA	1.4	0.9–1.8						
BMI			1.05	0.97–1.13	0.244			
BSA			0.1	0.01–2.02	0.132			

*IQR* interquartile range, *CR* complete response, *PR* partial response, *SD* stable disease, *PD* progressive disease, *NA* not assessed, *HR* hazard ratio, *CI* confidence interval, *BMI* body mass index, *BSA* body surface area, *PFS* progression-free survival, *AE* adverse events

^a^There is one missing data point on smoking status

A multivariate analysis was further performed to determine the independent prognostic factors for PFS. Liver metastases (vs. no liver metastases, HR: 2.17, 95% CI: 1.11–4.26, *p* = 0.023) and tumor responses of CR/PR (vs. PD/NA, HR: 0.05, 95% CI: 0.02–0.13, *p* < 0.001) and SD (vs. PD/NA, HR: 0.05, 95% CI: 0.01–0.19, *p* < 0.001) were identified as independent prognostic factors for PFS (Table 2).

### Prognostic factors for OS

A univariate analysis was performed to identify the possible prognostic factors for OS in patients treated with afatinib. Patients with a starting afatinib dose of 40 mg (vs. 30 mg, mOS: 17.5 vs. 8.1 months, HR: 0.59, 95% CI: 0.34–1.05, *p* = 0.073) showed favorable OS (Fig. 2B). Patients who had liver metastases (vs. no liver metastases, mOS: 3.1 vs. 13.8 months, HR: 1.85, 95% CI: 0.97–3.52, *p* = 0.061) showed unfavorable OS (Fig. 2D). Patients who achieved CR/PR (vs. PD/NA, mOS: 18.1 vs. 1.4 months, HR: 0.15, 95% CI: 0.08–0.3, *p* < 0.001) or SD (vs. PD/NA, mOS: 20.3 vs. 1.4 months, HR: 0.14, 95% CI: 0.05–0.39, *p* < 0.001) had better OS than those who had PD/NA (Fig. 2F).

Because only tumor response was significant on univariate analysis, those prognostic factors with *p* < 0.1 were included in the multivariate analysis to identify potential independent prognostic factors for OS. Liver metastases (vs. no liver metastases, HR: 2.15, 95% CI: 1.1–4.18, *p* = 0.024) and tumor responses of CR/PR (vs. PD/NA, HR: 0.14, 95% CI: 0.05–0.42, *p* < 0.001) and SD (vs. PD/NA, HR: 0.14, 95% CI: 0.07–0.30, *p* < 0.001) were independent prognostic factors for OS (Table 3).

**Table 3:** Univariate and multivariate analysis of prognostic factors in patients (OS)

Parameters	Univariate					Multivariate		
	Median (months)	95% CI	HR	95% CI	*p*-value	HR	95% CI	*p*-value
Age								
≤ 65	15.8	10.3–21.4	0.91	1.59–0.52	0.744			
> 65	8.2	1.1–15.3						
Sex								
Male	13.3	3.8–22.9	0.77	0.43–1.38	0.375			
Female	11.6	5.3–17.8						
Smoking status								
Smoker	22.3	16.7–27.9	0.62	0.28–1.39	0.244			
Never smoker	11.3	5.5–17.0						
Performance status, PS								
PS 2	15.8	8.8–22.9	0.86	0.49–1.53	0.615			
PS ¾	8.4	2.3–14.6						
Mutation					0.52			
L858R	11.7	7.7–15.6	0.67	0.27–1.65	0.382			
19del	18.1	9.1–27.1	0.59	0.24–1.46	0.253			
Uncommon	3.5	0.5–6.6						
Starting dose								
40 mg	17.5	11.6–23.4	0.59	0.34–1.05	0.073	0.93	0.51–1.72	0.827
30 mg	8.1	2.6–13.5						
Metastatic sites								
Lung								
Yes	10.1	4.9–15.2	1.26	0.72–2.19	0.422			
No	15.8	11.0–20.7						
Liver								
Yes	3.1	0.0–18.5	1.85	0.97–3.52	0.061	2.15	1.1–4.18	0.024
No	13.8	8.9–18.6						
Brain								
Yes	17.5	11.6–23.4	0.94	0.54–1.64	0.838			
No	10.8	6.7–15.0						
Bone								
Yes	18.1	11.5–24.8	0.66	0.38–1.15	0.147			
No	8.4	1.7–15.2						
Pleura								
Yes	8.1	0.0–17.8	1.59	0.90–2.81	0.112			
No	15.5	8.2–22.8						
Adrenal gland								
Yes	10.1	0.0–29.7	1.28	0.46–3.55	0.641			
No	12.9	8.4–17.3						
Distant lymphadenopathy								
Yes	15.5	0.0–39.8	1.11	0.47–2.62	0.804			
No	11.7	8.1–15.2						
Dose reduction/interruption								
Yes	20.3	15.8–24.8	0.65	0.37–1.15	0.138			
No	8.1	0.1–16.1						
Discontinuation, AE-related								
Yes	17.5	0.0–38.6	0.59	0.23–1.48	0.261			
No	11.7	8.2–15.2						
Clinical tumor response					< 0.001			< 0.001
CR/PR	18.1	13.6–22.7	0.15	0.08–0.3	< 0.001	0.14	0.05–0.42	< 0.001
SD	20.3	0.0–43.5	0.14	0.05–0.39	< 0.001	0.14	0.07–0.30	< 0.001
PD/NA	1.5	0.0–3.1						
BMI			1.01	0.93–1.10	0.867			
BSA			0.33	0.02–5.99	0.451			

*CR* complete response, *PR* partial response, *SD* stable disease, *PD* progressive disease, *NA* not assessed, *AE* adverse events, *BMI* body mass index, *BSA* body surface area, *HR* hazard ratio, *CI* confidence interval, *OS* overall survival

### Predictive factors for tumor response

In this study, patients with SD had comparable survival outcomes as patients with CR/PR; therefore, achieving durable disease control rather might be more important for patients with poor PS than a profound objective response. Therefore, potential predictors of disease control were investigated (Table 1). Bone metastasis (DCR: 81.3% vs 56.7%, *p* = 0.036) and dose reduction/interruption due to AEs (DCR: 87.0% vs. 59.0%, *p* = 0.021) were the only predictors for disease control identified in this cohort. Patients with a starting dose of 40 mg (DCR for 40 mg vs. 30 mg: 76.9% vs. 56.5%, *p* = 0.092), lower BMI (22.1 vs. 25.3, *p* = 0.063), and younger age (median age: 62.8 years in the DCR group and 74.3 years in the PD/NA group, *p* = 0.091; DCR for patients < 65 years vs. ≥ 65 years, 81.5% vs.. 60.0%, *p* = 0.069) trended to have more disease control than those without these features.

### Adverse events and association with the starting dose of afatinib

Diarrhea (87.1%) was the most frequently reported AE, followed by skin rashes (62.9%), paronychia (45.2%), and mucositis/stomatitis (38.7%). Most reported AEs were mild (Grade 1/2) and manageable. Among severe (Grade ≥ 3) AEs, diarrhea (11.3%) remained the most frequently reported AE, followed by paronychia (9.7%), mucositis/stomatitis (6.5%), and skin rashes (4.8%). Overall, treatment with 40 mg afatinib was more likely to be associated with the occurrence of all grades of AEs (100% vs 91.3%, *p* = 0.061) and severe AEs (30.8% vs 8.7%, *p* = 0.045) than treatment with 30 mg. For specific AEs, such as diarrhea, paronychia, skin lesions, and mucositis, treatment with 40 mg afatinib was associated with a higher incidence of AEs than treatment with 30 mg afatinib, although this difference was not significant, likely due to the limited number of cases (Table 4).

**Table 4:** Treatment-related AEs

AEs	Any grade							Grade 3–4						
	All (*N* = 62)	%	Afatinib 40 mg (*N* = 39)	%	Afatinib 30 mg (*N* = 23)	%	*p*-value	All (*N* = 62)	%	Afatinib 40 mg (*N* = 39)	%	Afatinib 30 mg (*N* = 23)	%	*p*-value
Any adverse effects	60	96.8%	39	100.0%	21	91.3%	0.061	14	22.6%	12	30.8%	2	8.7%	0.045
Diarrhea	54	87.1%	36	92.3%	18	78.3%	0.111	7	11.3%	6	15.4%	1	4.3%	0.185
Acneiform	8	12.9%	6	15.4%	2	8.7%	0.448	0	0.0%	0	0.0%	0	0.0%	–
Paronychia	28	45.2%	20	51.3%	8	34.8%	0.207	6	9.7%	5	12.8%	1	4.3%	0.276
Skin lesions	39	62.9%	28	71.8%	11	47.8%	0.059	3	4.8%	3	7.7%	0	0.0%	0.173
Pruritus	1	1.6%	1	2.6%	0	0.0%	0.439	0	0.0%	0	0.0%	0	0.0%	–
Nausea and vomiting	5	8.1%	3	7.7%	2	8.7%	0.889	0	0.0%	0	0.0%	0	0.0%	–
Constipation	3	4.8%	3	7.7%	0	0.0%	0.173	0	0.0%	0	0.0%	0	0.0%	–
Dry skin	5	8.1%	2	5.1%	3	13.0%	0.269	0	0.0%	0	0.0%	0	0.0%	–
Mucositis	24	38.7%	18	46.2%	6	26.1%	0.117	4	6.5%	4	10.3%	0	0.0%	0.112
Hand foot syndrome	2	3.2%	2	5.1%	0	0.0%	0.270	1	1.6%	1	2.6%	0	0.0%	0.439
Eye	2	3.2%	2	5.1%	0	0.0%	0.270	0	0.0%	0	0.0%	0	0.0%	–
Edema	1	1.6%	1	2.6%	0	0.0%	0.439	0	0.0%	0	0.0%	0	0.0%	–
Onycholysis	1	1.6%	1	2.6%	0	0.0%	0.439	0	0.0%	0	0.0%	0	0.0%	–

*AE* adverse event

### Subsequent treatment after afatinib

Overall, 25 (40.3%) patients received subsequent treatment after afatinib failure, including chemotherapy (*n* = 14), TKIs other than osimertinib (*n* = 14), osimertinib (*n* = 4), bevacizumab (*n* = 3), and immune checkpoint inhibitors (*n* = 2). No significant association was found between the starting afatinib dose, tumor response, and subsequent treatment; however, patients with PD/NA were more likely to receive subsequent immunotherapy (10.5% vs. 0.0%, *p* = 0.031), which was not clinically significant because only 2 of 62 patients received subsequent immunotherapy (Table 5).

**Table 5:** Subsequent treatments

**Subsequent treatment**	Overall (*N* = 62)	%	Starting dose					Clinical response				
			Afatinib 40 mg (*N* = 39)	%	Afatinib 30 mg (*N* = 23)	%	*p*-value	CR/PR/SD (*N* = 47)	%	PD/NA (*N* = 15)	%	*p*-value
Yes	25	40.3%	15	38.5%	10	43.5%	0.697	20	42.6%	5	33.3%	0.526
No	37	59.7%	24	61.5%	13	56.5%		27	57.4%	10	66.7%	
**Patients receiving subsequent treatment**	Overall (*n* = 25)	%	Afatinib 40 mg (*N* = 39)	%	Afatinib 30 mg (*N* = 23)	%	*p*-value	CR/PR/SD (*N* = 47)	%	PD/NA (*N* = 15)	%	*p*-value
Chemotherapy												
Yes	14	56.0%	7	46.7%	7	70.0%	0.256	11	23.4%	3	20.0%	0.784
No	11	44.0%	8	53.3%	3	30.0%		36	76.6%	12	80.0%	
TKI other than osimertinib												
Yes	14	56.0%	9	60.0%	5	50.0%	0.903	11	23.4%	3	20.0%	0.784
No	11	44.0%	6	40.0%	5	50.0%		36	76.6%	12	80.0%	
Osimertinib												
Yes	4	16.0%	2	13.3%	2	20.0%	0.581	3	6.4%	1	6.7%	0.969
No	21	84.0%	13	86.7%	8	80.0%		44	93.6%	14	93.3%	
Bevacizumab												
Yes	3	12.0%	2	13.3%	1	10.0%	0.89	3	6.4%	0	0.0%	0.316
No	22	88.0%	13	86.7%	9	90.0%		44	93.6%	15	100.0%	
Immune checkpoint inhibitors												
Yes	2	8.0%	1	6.7%	1	10.0%	0.701	1	2.1%	1	6.7%	0.386
No	23	92.0%	14	93.3%	9	90.0%		46	97.9%	14	93.3%	

*TKI* tyrosine kinase inhibitor, *CR* complete response, *PR* partial response, *SD* stable disease

## Discussion

To the best of our knowledge, this is the largest cohort study to investigate the feasibility and efficacy of afatinib treatment for EGFRm+ NSCLC patients with PS ≥ 2 because these patients have been excluded from previous clinical trials. This real-world experience from a single institute demonstrated that afatinib was effective and well-tolerated among patients with poor PS. The ORR, DCR, mPFS, and mOS were 58.1, 69.4%, 8.8 months, and 12.9 months, respectively. Dose modification and discontinuation frequently occurred but did not compromise clinical benefits. Treatment-related AEs frequently occurred, but most were mild and tolerable. In addition, liver metastasis and clinical tumor response were identified as independent prognostic factors for PFS and OS. Furthermore, bone metastasis and dose modification (reduction/interruption) were the only predictors for disease control. This study provided additional evidence to support the use of afatinib in patients with poor PS.

The major causes of poor PS in NSCLC patients were underlying comorbidities and disseminated/advanced NSCLC; therefore, these patients may only have one line of treatment option and might not receive subsequent treatment if their tumors do not respond to afatinib treatment. In our analysis, clinical tumor response was the most important prognostic factor for survival, and patients who did not achieve disease control after afatinib treatment had a worse prognosis (mPFS: 1.4 months, mOS: 1.5 months). In addition, patients who developed metastasis at specific sites were associated with shorter mPFS and mOS, except those with bone and brain metastases (Tables 2 and 3 and Fig. 3). This outcome could be explained by the finding that afatinib is effective for patients with bone and brain metastases [18]; therefore, poor PS caused by bone and brain metastases may be reversed by afatinib. Systemic treatments other than TKIs are effective in patients with good PS without brain metastasis; however, in the absence of brain metastasis, the outcomes of patients with poor PS in our study might not be comparable to that of patients with good PS in other studies [18]. This difference may be due to poor PS (which is a result of greater toxicity and lower response rates) undermining the efficacy of subsequent systemic treatment.

A large cohort study based on the Taiwan Cancer Registry, conducted in 2011–2015, compared the efficacy of three different TKIs—gefitinib, erlotinib, and afatinin. The results showed a time to treatment failure (TTF) and an OS of 15.8 months and not reached, respectively, for afatinib treatment during the median follow-up period of 17.3 months [21]. Median event-free survivals indicated the time when 50% of patients had reached the events. If less than 50% of the patients were alive during the follow-up, this would be recorded as “not reach.” Seventy-three of seven hundred fifty-one patients who received afatinib had a PS of 2, and those with PS > 2 were excluded from this study. A PS of 2 was an independent prognostic factor for TTF and OS, but detailed data were not available. In addition, data on response evaluation, PFS, AEs, *EGFR* mutation type, and tumor involvement were not reported, which are the major limitations of retrospective cohort studies performed using data from the Cancer Registry databank.

Liver metastasis was an independent unfavorable prognostic factor. Patients with liver metastasis had worse survival outcomes (mPFS: 3.1 months and mOS: 3.1 months) compared with patients without liver metastases, which agreed with previous studies. A study demonstrated that the mPFS was only 2.3 months in EGFRm+ patients with liver metastases treated with erlotinib as second- or third-line treatment [22]. Another study reported that liver metastasis was a significantly poor prognostic factor, with mPFS and mOS of 5.9 and 11.9 months, respectively, in EGFRm+ patients with liver metastases treated with first-line TKIs [23].

In clinical practice, physicians usually prescribe a fixed dose of TKI therapy rather than a formulated dose as is used in chemotherapy. In this study, the starting dose of 40 mg was associated with good disease control and favorable PFS and OS. In addition, AE-related dose modification (reduction/interruption) was a predictor of better disease control and longer mPFS and mOS. Furthermore, patients whose tumors achieved disease control had a trend toward lower BMI than those who did not achieve disease control (22.1 vs. 25.3, *p* = 0.063). These findings indicated that dose and BMI might be used to predict the clinical benefit and associated AEs; therefore, the association among survival outcomes, dose, and BMI should be investigated in a larger cohort.

The frequency of AEs may be underestimated in this study due to its retrospective nature; however, the frequency of AEs in this study was much higher than those reported in previous real-world studies [19, 24]. Although 37.1% of patients required dose modifications and 12.9% required treatment discontinuation, their survival outcomes were not affected by treatment interruption. Furthermore, patients with dose modifications showed trends toward longer PFS and OS compared with those without dose modifications. This finding suggested that dose modification in patients who were intolerant to AEs did not compromise the treatment outcomes of those patients. Patients who discontinued afatinib treatment typically received first-generation TKIs, such as gefitinib, as a subsequent treatment, which have been associated with fewer AEs than afatinib [9].

Patients with an initial 40 mg dose had longer PFS (*p* = 0.043) and OS (*p* = 0.073) than those who started at 30 mg. However, this finding was not considered significant in the multivariate analysis. In addition, treatment with 40 mg afatinib induced more AEs than treatment with 30 mg afatinib in the current cohort. In a study of 48 patients, those who received 30 mg daily as the initial dose tended to be older, female, and have a smaller body size [25]. Patients with an initial dose of 40 mg afatinib daily showed no significant differences in ORR, DCR, or PFS compared with those who started with an initial dose of 30 mg afatinib daily. Patients receiving 30 mg daily had a significantly lower incidence of diarrhea than those receiving 40 mg daily (41% vs. 100%, *p* < 0.0001) [25]. Other studies also reported no differences in clinical outcomes between 30 and 40 mg doses of afatinib [12, 26].

The retrospective nature of this study and the limited number of patients were the major study limitations; however, to the best of our knowledge, this study represents the largest cohort study enrolling patients with PS ≥ 2 receiving afatinib. The causes of poor PS were difficult to determine, which represents one of the limitations of a retrospective study. Some patients did not undergo tumor evaluation because their disease was not well-controlled after afatinib initiation. Although AEs may be difficult to be recorded accurately due to the retrospective nature of the study, the overall AE frequency was comparable with the rates reported in previous clinical trials [7–9]. Moreover, 96.8% of the patients in these studies experienced various grades of AE, indicating that the results of the current study are reliable.

In conclusion, we reported the real-world experience of afatinib when used as first-line treatment for EGFRm+ NSCLC patients with poor PS. This study demonstrated that afatinib is feasible and effective for EGFRm+ NSCLC patients classified as poor PS, is generally well-tolerated, and has acceptable anti-tumor activity. This study provided evidence to support the use of afatinib to treat patients with poor PS. However, further studies with a larger cohort remain necessary to confirm our findings.

## Data Availability

The data supporting the findings of this study are available from Cancer Registry databank at CGMH and Chang Gung Research Database [20], but restrictions apply to their availability. These data were used under the approval of Institutional Review Board at CGMH for the current study, and so are not publicly available. The de-linked, and anonymized datasets used and/or analyzed during the current study are available from the corresponding author on reasonable request.

## Abbreviations

AE: Adverse events
BMI: Body mass index
BSA: Body surface area
CI: Confidence interval
CR: Complete response
DCR: Disease control rate
ECOG: Eastern Cooperative Oncology Group
EGFR: Epidermal growth factor receptor
EGFRm+: EGFR-mutation-positive
HER: Human epidermal growth factor receptor
HR: Hazard ratio
mOS: Median OS
mPFS: Median PFS
NA: Not assessed
NSCLC: Non-small-cell lung cancer
OS: Overall survival
PD: Progressive disease
PFS: Progression-free survival
PR: Partial response
PS: Performance status
SD: Stable disease
TTF: Time to treatment failure
